# Reactivation of Choroidal Neovascularization in Bilateral Punctate Inner Choroidopathy: A Case Report

**DOI:** 10.7759/cureus.112681

**Published:** 2026-07-14

**Authors:** Nurul Syafiela Zainuddin, Norakmal Bahari, Safinaz Mohd Khialdin

**Affiliations:** 1 Ophthalmology, Hospital Kuala Lumpur, Kuala Lumpur, MYS; 2 Ophthalmology, Faculty of Medicine, Universiti Kebangsaan Malaysia, Kuala Lumpur, MYS

**Keywords:** anti-vascular endothelial growth factor (anti-vegf) therapy, choroidal neovascularization, immunosuppressant, punctate inner choroidopathy, recurrence

## Abstract

Punctate inner choroidopathy (PIC) is an idiopathic inflammatory chorioretinopathy predominantly affecting young myopic women. While often self-limiting, PIC can be complicated by choroidal neovascularization (CNV), leading to significant visual impairment. We report a case of a 50-year-old woman with bilateral PIC who developed reactivation of CNV in the right eye despite ongoing immunosuppressive therapy. Management with intravitreal ranibizumab and systemic corticosteroids resulted in favorable anatomical and visual outcomes. This case underscores the importance of vigilant monitoring and a multimodal treatment approach in managing PIC complicated with CNV.

## Introduction

Punctate inner choroidopathy (PIC) is a rare, idiopathic inflammatory condition characterized by multiple small, yellow-white lesions at the level of the inner choroid and retinal pigment epithelium (RPE), typically affecting young myopic women [1]. Although often self-limiting, PIC can be complicated by choroidal neovascularization (CNV), which poses a risk of permanent vision loss [2]. The pathogenesis of CNV in PIC is thought to involve inflammatory damage to the RPE and Bruch’s membrane, resulting in an angiogenic stimulus [3]. Anti-vascular endothelial growth factor (anti-VEGF) agents have become the mainstay treatment for CNV in PIC, often in conjunction with systemic immunosuppression to control underlying inflammation [4-9]. Herein, we present a case of bilateral PIC with reactivation of CNV in the right eye, despite systemic immunosuppressive agents.

## Case presentation

A 50-year-old woman with myopia and a known history of bilateral PIC diagnosed five years earlier presented with a two-week history of progressive visual disturbances in the right eye, described as metamorphopsia and a central scotoma. She had previously been treated for CNV in the right eye at a private eye center with a single intravitreal injection of ranibizumab, which resulted in complete resolution of subretinal fluid, with baseline optical coherence tomography (OCT) demonstrating no disease activity thereafter. She was only prescribed oral prednisolone 10 mg daily for six months of maintenance treatment; however, she was non-compliant due to weight gain. She denied any systemic symptoms, and her past medical and family histories were unremarkable.

**Figure 1:**
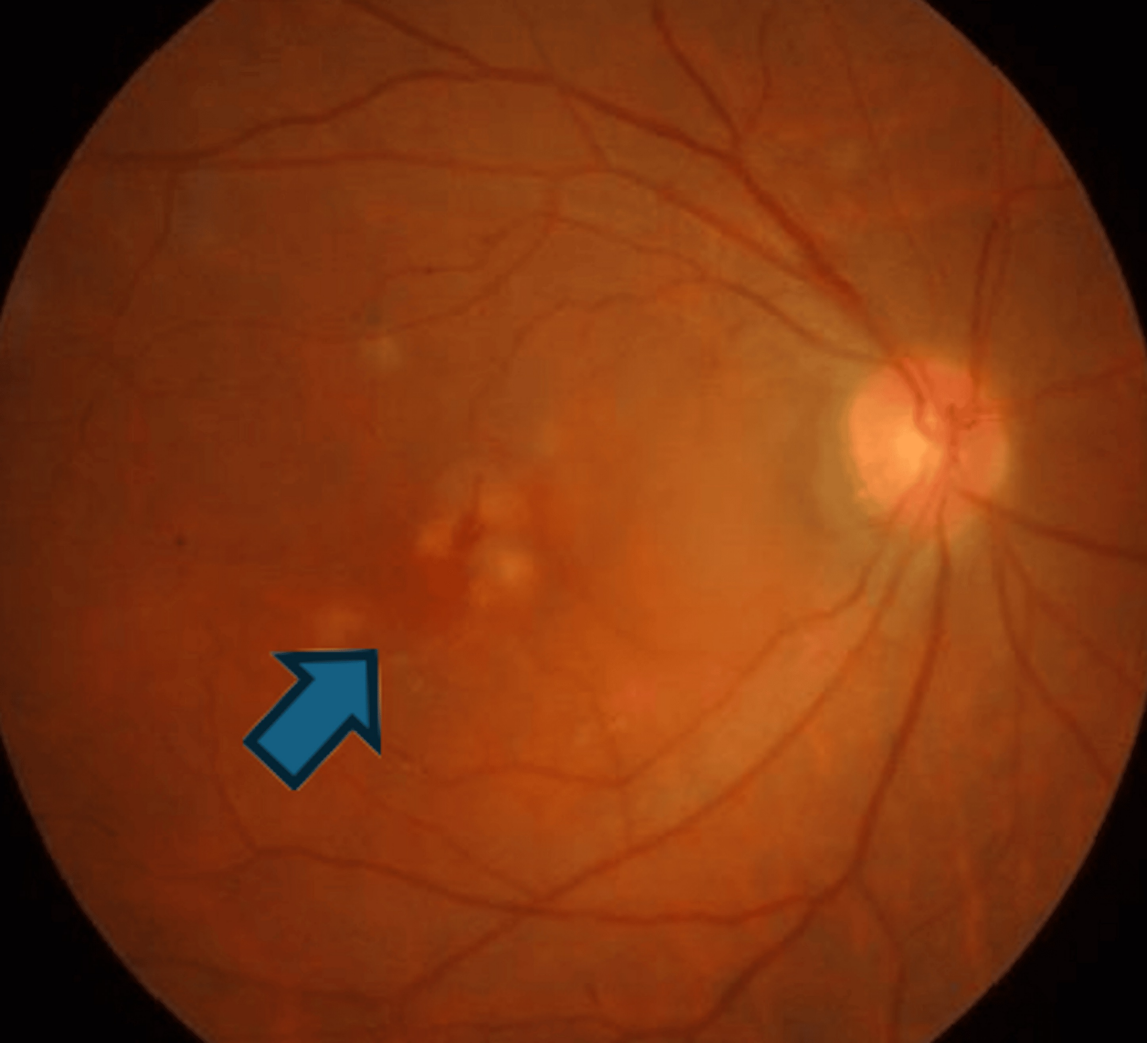
Color fundus photography of the patient’s right eye showing multiple yellowish white lesion and subretinal hemorrhage.

**Figure 2:**
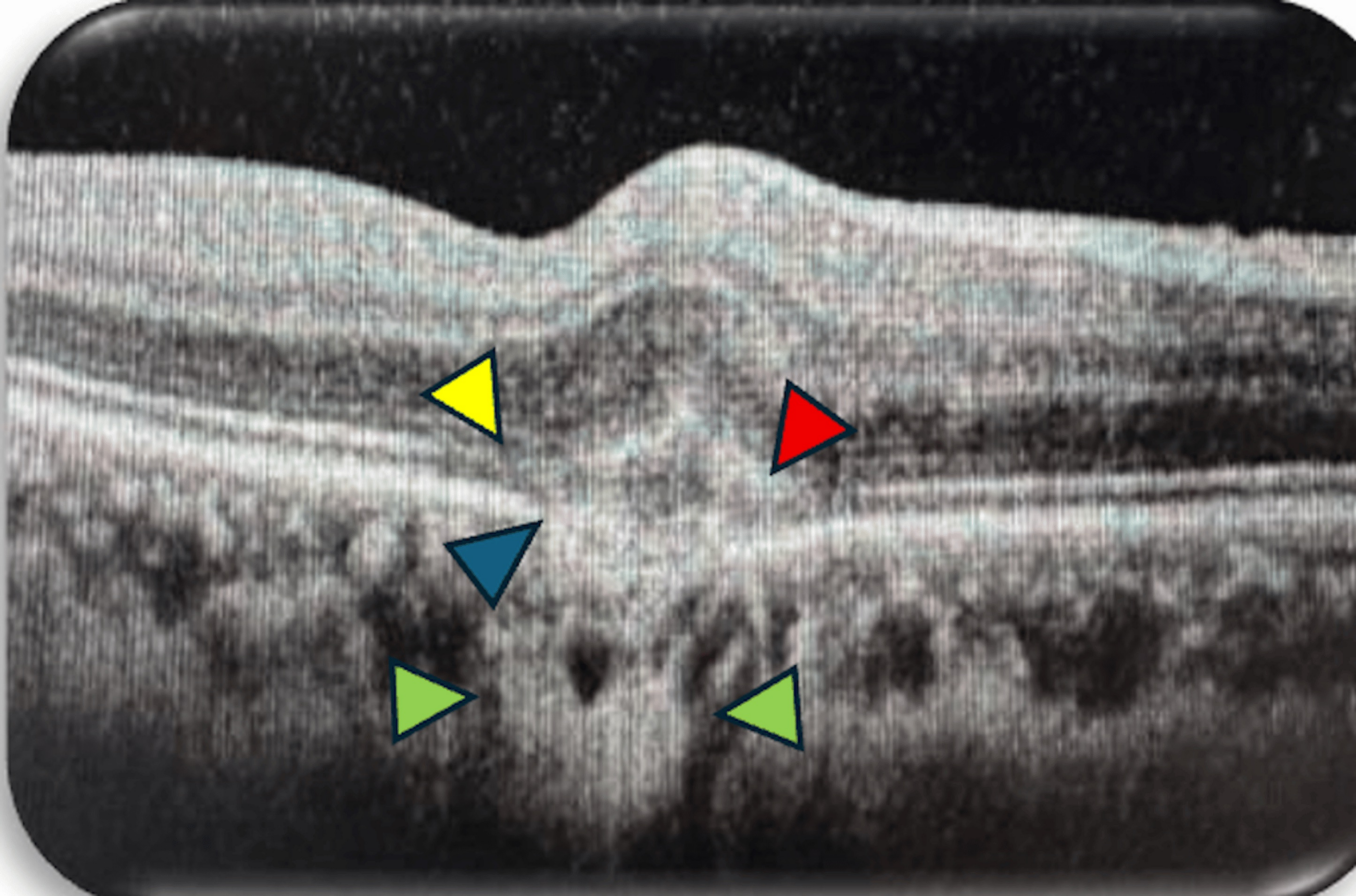
Optical coherence tomography showing disruption of the ellipsoid zone (yellow arrowhead), outer retinal fuzziness (red arrowhead), hyperreflective choroidal back-shadowing (green arrowhead), and disruption of the basement membrane (blue arrowhead).

On examination, her best-corrected visual acuity (BCVA) was 6/12 in the right eye and 6/9 in the left eye. Intraocular pressures were within normal limits. Anterior segment examination was unremarkable, with no evidence of anterior chamber inflammation. Posterior segment evaluation revealed bilateral multifocal yellowish choroidal lesions, with a small subretinal hemorrhage superior to the macula in the right eye (Figure 1). There were no vitreous cells or vitreous haze. OCT of the right eye (Figure 2) showed subfoveal focal choroidal excavation with overlying RPE elevation and disappearance of the photoreceptor layer. OCT of the left eye showed RPE irregularities and choroidal excavation. An OCT diagnosis of bilateral PIC complicated by CNV in the right eye was made.

**Figure 3:**
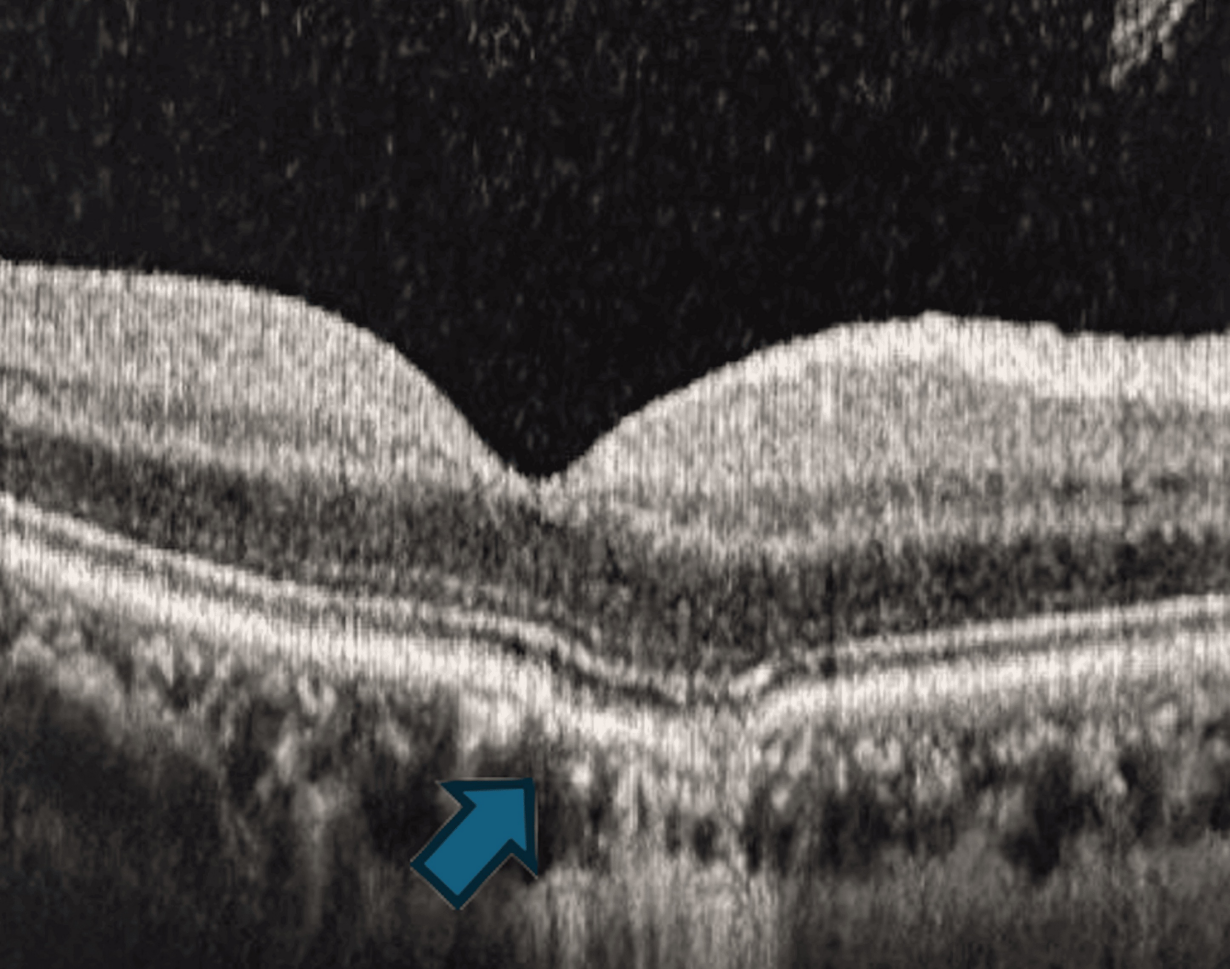
Post-treatment optical coherence tomography showed restoration of the outer retina layer and ellipsoid zone, flattening of retinal pigment epithelium elevation, and absence of subretinal fluid.

The patient was treated with intravitreal ranibizumab 0.5 mg in the right eye, and the dose of oral corticosteroids was increased to 1 mg/kg with a gradual taper over six weeks. Counseling and reassurance regarding steroid use were provided, and the patient was willing to continue treatment. She was also started on mycophenolate mofetil for the prevention of recurrence. At serial follow-up visits, the patient reported subjective improvement in visual symptoms. Post-treatment OCT macula (Figure 3) revealed the resolution of RPE elevation with the reappearance of the photoreceptor layer. The patient initially received monthly intravitreal ranibizumab injections in the right eye due to persistent, although reduced, CNV activity. Subsequently, the injection interval was tailored according to disease activity. In total, she received three doses of intravitreal ranibizumab.

Her right eye remained stable, with no recurrence of CNV or development of new inflammatory lesions. Final BCVA improved to 6/6 in both eyes three months after her last dose of intravitreal ranibizumab. The patient was maintained on low-dose oral prednisolone 5 mg daily for an additional three months before discontinuation. Her second-line immunosuppressive agent, mycophenolate mofetil 1 g twice daily, was continued. No significant adverse effects were noted during treatment.

## Discussion

This case highlights the occurrence of recurrent CNV in bilateral PIC and underscores the importance of early recognition, prompt treatment, and long-term follow-up. Although PIC typically affects younger individuals [1]. This case demonstrates that CNV may recur despite clinically quiescent PIC and the absence of overt intraocular inflammation, underscoring the importance of long-term follow-up and regular multimodal imaging for early detection and timely intervention. 

The development of CNV in PIC is believed to result from a breakdown of the RPE and Bruch’s membrane due to chronic inflammation, facilitating the ingrowth of neovascular membranes [2, 3]. CNV in PIC can be challenging to detect clinically, especially when subretinal hemorrhage is minimal. Thus, imaging modalities such as OCT and fundus fluorescein angiography (FFA) are valuable for the diagnosis and monitoring of PIC-associated CNV. In this patient, the diagnosis and follow-up were based on characteristic OCT findings, and FFA was not considered necessary because the clinical and OCT features were sufficient to guide management [4].

The mainstay of CNV treatment in inflammatory chorioretinopathies is intravitreal anti-VEGF therapy. Ranibizumab, bevacizumab, and aflibercept have all shown favorable outcomes in PIC-associated CNV, with no clear evidence of superiority among them [5, 6]. Most patients require one to three injections, depending on disease activity. In our patient, three ranibizumab injections effectively resolved CNV and restored visual acuity.

In addition to anti-VEGF therapy, controlling the underlying inflammatory process is essential to prevent recurrence. Systemic corticosteroids provide rapid control of inflammation, while steroid-sparing immunosuppressants such as mycophenolate mofetil are beneficial for long-term disease suppression and reducing steroid-associated side effects [7-9]. In this patient, she developed weight gain after starting steroids; hence, the patient is non-compliant with oral prednisolone therapy. This may contribute to the development of recurrence of CNV. However, this patient’s long-term stability without CNV recurrence highlights the role of continued immunomodulatory therapy in maintaining remission.

Another consideration is the importance of patient education and adherence to follow-up. This patient recognized the early signs of CNV recurrence and sought medical attention promptly, facilitating timely intervention. Regular monitoring with OCT and clinical evaluation is crucial, especially in patients with a prior history of CNV or active inflammatory lesions.

## Conclusions

This case highlights the clinical challenge of managing CNV in PIC, particularly in patients with a history of prior CNV and ongoing immunosuppression. A combination of anti-VEGF injections and systemic immunomodulation resulted in favorable outcomes in terms of visual acuity and disease control. Clinicians should maintain a high index of suspicion for CNV in patients with PIC who present with new visual symptoms and utilize multimodal imaging and tailored therapy to optimize patient outcomes.
